# Letter to editor regarding ‘Dexmedetomidine nasal spray for patients undergoing endoscopic retrograde cholangiopancreatography: protocol for a randomized controlled trial’

**DOI:** 10.1080/07853890.2026.2635199

**Published:** 2026-02-24

**Authors:** Riya Kalra, Kanu Goyal, Manu Goyal

**Affiliations:** Maharishi Markandeshwar Institute of Physiotherapy and Rehabilitation, MM(DU), Mullana, Haryana, India

Dear Editor

We read with great interest the recently published protocol by Zhuang et al. *‘Dexmedetomidine nasal spray for patients undergoing Endoscopic retrograde cholangiopancreatography: protocol for a randomized controlled trial.’* The authors deserve appreciation for focusing on an important and often challenging aspect of endoscopic retrograde cholangiopancreatography (ERCP)—achieving effective sedation while minimising cardiorespiratory complications [1].

ERCP is well known to cause significant discomfort and physiological stress, and the search for safer, more patient-friendly sedation strategies remain clinically relevant. In this context, the use of intranasal dexmedetomidine as an adjunct to propofol is both thoughtful and innovative. The non-invasive route, combined with the favourable pharmacological profile of dexmedetomidine, makes this approach particularly appealing in routine clinical practice [2].

We found the study methodology to be carefully planned, with appropriate randomization, blinding, and adherence to SPIRIT guidelines. The choice of total propofol consumption as the primary outcome is clinically meaningful, as lower sedative requirements may reduce the incidence of hypotension, hypoxemia, and delayed recovery. The inclusion of post-procedural fatigue as a secondary outcome is also commendable, as it reflects a growing emphasis on patient-centred recovery rather than procedural success alone.

That said, a few aspects warrant consideration. Firstly, as a single-centre study, the findings may have limited generalizability, particularly across settings with varying sedation practices [3]. Secondly, excluding patients above 70 years of age, while understandable from a safety perspective, may limit applicability to an older population that commonly undergoes ERCP and may stand to benefit significantly from optimized sedation techniques. Future multicentre studies with broader inclusion criteria could help strengthen the evidence base. Additionally, the protocol targets deep sedation (MOAA/S scores of 1–2), which is inherently associated with higher risks of respiratory and haemodynamic compromise. It remains unclear whether intranasal dexmedetomidine could support adequate procedural conditions at lighter sedation levels, potentially improving safety. Assessing fatigue only 15 min after emergence may also be insufficient to capture meaningful recovery, particularly in older or ambulatory patients [4].

The use of a composite secondary outcome combining hypotension and hypoxemia, while pragmatic, may obscure the individual incidence and clinical relevance of each complication. Furthermore, excluding patients who require tracheal intubation from the primary efficacy analysis may underestimate sedation-related difficulties encountered in real-world practice. Finally, the fixed dosing approach does not explore whether individualized dosing strategies could influence outcomes

It may also be interesting for future research to explore the assessment of long-term recovery parameters and patient satisfaction beyond the immediate post-procedure period, which could add further clinical value [5].

Overall, this protocol represents a well-designed and promising study that addresses a relevant clinical question. We look forward to the results and believe they will contribute meaningfully to improving sedation practices for ERCP

## Data Availability

Data sharing does not apply to this article as no new data were created or analysed in this study.
